# Determination Quantification of Molecular Interactions in Protein Films: A Review

**DOI:** 10.3390/ma7127975

**Published:** 2014-12-10

**Authors:** Felicia Hammann, Markus Schmid

**Affiliations:** 1 Fraunhofer-Institute for Process Engineering and Packaging IVV, Giggenhauser Strasse 35, Freising 85354, Germany; E-Mail: feeha@mytum.de; 2 Chair of Food Packaging Technology, Technische Universität München, Weihenstephaner Steig 22, Freising 85354, Germany

**Keywords:** whey protein, soy protein, wheat gluten, cross-linking, SDS‑PAGE, NMR, CD, FTIR, protein solubility study

## Abstract

Protein based films are nowadays also prepared with the aim of replacing expensive, crude oil-based polymers as environmentally friendly and renewable alternatives. The protein structure determines the ability of protein chains to form intra- and intermolecular bonds, whereas the degree of cross-linking depends on the amino acid composition and molecular weight of the protein, besides the conditions used in film preparation and processing. The functionality varies significantly depending on the type of protein and affects the resulting film quality and properties. This paper reviews the methods used in examination of molecular interactions in protein films and discusses how these intermolecular interactions can be quantified. The qualitative determination methods can be distinguished by structural analysis of solutions (electrophoretic analysis, size exclusion chromatography) and analysis of solid films (spectroscopy techniques, X-ray scattering methods). To quantify molecular interactions involved, two methods were found to be the most suitable: protein film swelling and solubility. The importance of non-covalent and covalent interactions in protein films can be investigated using different solvents. The research was focused on whey protein, whereas soy protein and wheat gluten were included as further examples of proteins.

## 1. Introduction

Packaging material fulfils numerous functions. Most importantly it maintains the quality and safety of packed foods. The quality of the food has to be ensured during the whole life cycle, this includes transportation and storage at the retailors and customers. To provide safe foodstuffs it is necessary to protect them depending on their respective requirements against oxygen, water vapor, contamination, and physical, chemical, or biological damage [1]. To achieve these requirements multilayer films are widely used in the food packaging sector [1]. Multilayer films combine the different advantages of each single layer. One example is ethylene vinyl alcohol (EVOH), which is embedded in other polyolefins and/or polyesters, providing a high oxygen barrier. However, EVOH is neither renewable nor biodegradable [2,3].

A research on films made from proteins is driven by the demand for environmentally friendly, renewable polymeric materials. One objective faced by the plastics industry is to replace petroleum-based polymer layers in packaging films, such as EVOH, with biodegradable and renewable raw materials with equal or improved properties. In recent years, films and coatings have been made from renewable sources, such as casein, whey, soy, corn zein, collagen, wheat gluten, keratin and egg albumin [4,5,6].

The formation of protein-based films is preceded in three steps. First, low-energy intermolecular bonds which stabilize polymers in the native state are relieved. Second, polymer chains are newly arranged and orientated. And third, the formation of a three dimensional network is stabilized by new interactions and bonds. A wet process based on dispersion or solubilization of proteins, and a dry process based on the thermoplastic processing of proteins are used to make protein-based films [4].

Protein-based films have been successfully made among others from whey protein isolate, soy protein isolate and wheat gluten [7,8,9,10,11,12,13,14,15,16]. Results have shown that proteins possess several functional properties for film formation. Although protein films have relatively poor water vapor barriers, due to their hydrophilic nature, they have excellent gas barrier properties [14,17,18,19,20,21,22,23,24]. A benefit of proteins is also their availability in high quantities. For example, whey protein is a by-product of cheese production, soy protein incurs in the soy oil extraction process, and wheat gluten is a by-product of starch fabrication [25,26,27]. A process of cross-linking is necessary to obtain coherent and flexible biofilms with improved mechanical and barrier properties. Furthermore, it is desirable that the biodegradability is maintained even if the films are cross-linked [24].

Therefore, the objective of this work was the identification of methods for determination and quantification of molecular interactions in protein films. In principle, the determination methods can be divided into the structural analysis of solutions and of solid films. The disadvantage of the cross-linking determination of solutions is that the samples must be dissolved. Possible consequences could be that interactions between the sample and dissolving agent change the protein conformation. 

This work focuses on determination methods of molecular interactions that are already widely used in connection with the three chosen proteins. Aside, recent methods such as NMR and X-ray scattering should be considered, but the findings are narrow.

## 2. Protein Films

### 2.1. Definition and Characteristics of Proteins

Proteins are organic macromolecules and built out of amino acids which are chained by peptide bonds. The primary structure is characterized by its sequential order of amino acids. Depending on the primary structure, the protein will assume different structures along the polymer chain. The geometry can be arranged as α-helix, β-sheet or as turns and is coiled and stabilized by hydrogen bonds, van der Waals, electrostatic, hydrophobic and disulfide interactions. The tertiary structure is the overall shape of a protein molecule. This structure is stabilized by intermolecular interactions of lateral chains. For a quarternary structure several protein molecules must be joined together to a functional protein complex. The protein structure determines the ability to interact with each other and with other film components [28,29].

The properties of proteins make them excellent starting materials for films and coatings. Intrinsic properties of the film components and extrinsic processing factors affect the final properties of the film [30]. The amino acid composition of the proteins is one crucial factor for intrinsic properties. The primary sequence of proteins is built up of charged, polar and nonpolar amino acids. This leads to a chemical potential along the protein chain. Resulting electrostatic interactions, hydrogen bonding, van der Waals forces and disulfide bridges can improve the stability of films [5,6,31,32]. Barone *et al*. [33] discovered that protein films are often more stable than polysaccharide-based ones due to intermolecular disulfide linkages which can be present in protein films.

Table 1 shows the amino acid building blocks of whey protein, soy protein and wheat gluten, which are commonly used already to make protein based films due to positive film forming properties and high potential concerning technofunctional properties. The thiol groups of cysteine residues allow intra- and intermolecular thiol-disulfide interchange, as noted for β-lactoglobulin [33]. A high amount of asparagine and glutamine residues is typical for storage proteins such as soy protein. A high number of glutamine turns wheat gluten into an acyl-donator for enzymatic cross-linking by transglutaminase. Both soy protein and wheat gluten are suited for intermolecular cross-linking by irradiation due to the relatively high content of aromatic acids (phenylalanine, tyrosine) [25,34,35,36]. More intrinsic factors of proteins are crystallinity, salt, hydrophobicity, surface charge, pKI, molecular size and three-dimensional structure. Processing temperature, drying condition, pH, ionic strength, relative humidity during processing and storage, shear and pressure are counted towards the extrinsic factors [25,37].

**Table 1:** Amino acid composition of β-lactoglobulin, β-conglycinin, γ-gliadins and whole gliadins according to [25,38,39,40,41].

Amino acid	β-lactoglobulin (Whey Protein) (mol%)	β-conglycinin (Soy Protein) (mol%)	γ-gliadins (Wheat Gluten) (mol%)	Whole Gliadins (Wheat Gluten) (per 100 g protein)
Alanine	5.4	4.0	2.3	28.6
Arginine	2.5	8.3	1.8	17.4
Asparagine	3.1	12.0	2.9	–
Aspartic acid	6.9	–	–	24.8
Cysteine	2.8	0.03	–	29.0
Glutamic acid	6.2	24.5	45.8	301.1
Glutamine	11.2	–	–	–
Glycine	0.9	3.5	1.4	26.8
Histidine	1.5	2.8	1.6	16.3
Isoleucine	6.3	4.5	4.4	38.0
Leucine	13.6	7.5	7.0	60.6
Lysine	10.5	6.1	–	5.0
Methionine	2.8	0.4	0.9.	10.9
Phenylalanine	3.2	5.4	5.2	37.5
Proline	4.2	4.7	14.5	142.0
Serine	3.3	5.4	4.3	53.6
Threonine	4.4	3.3	1.7	21.3
Tryptophan	2.0	–	–	3.8
Tyrosine	3.6	3.5	3.5	16.0
Valine	5.4	4.1	3.8	41.6

### 2.2. Whey Protein

**Figure 1:**
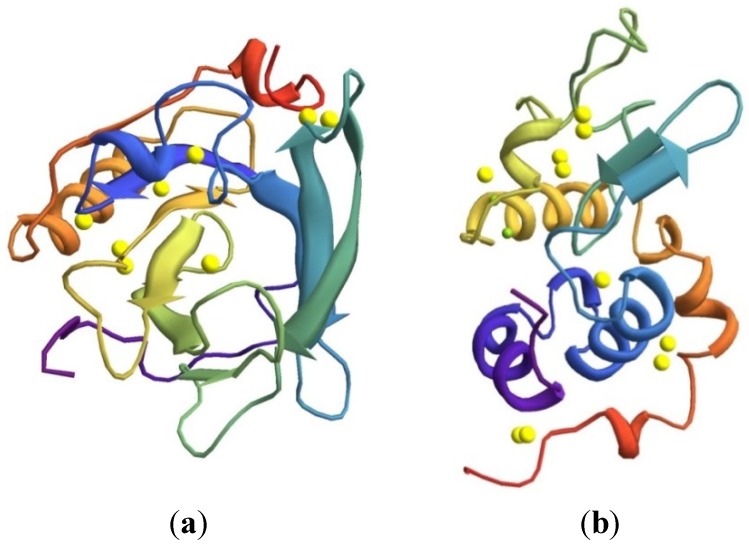
Tertiary structure of (**a**) β-lactoglobulin and (**b**) α-lactalbumin [52]. Figures are created with Wolfram Mathematica 9.0 based on protein data from [52].

Whey protein includes the following globular proteins: β-lactoglobulin, α-lactalbumin, bovine serum albumin, immunoglobulins and proteose peptones [42,43,44,45]. β-lactoglobulin is the major protein in whey (57% in whey protein [46]) and dominates the aggregation and gelation behavior of whey protein preparations. The primary structure of β-lactoglobulin consists of 162 amino acids and has a molecular weight of 18 kDa [44,47]. The secondary structure of β-lactoglobulin is dominated by beta-sheets and a three-turn alpha‑helix. The formation of two covalent disulfide bonds of four of the five cysteine molecules determines the tertiary structure of β-lactoglobulin (Figure 1) The disulfide bonds stabilize the globular quaternary structure, its nonpolar lateral chains are located in the molecule inside, and its polar lateral chain are positioned in the molecule outside. The remaining free sulfhydryl group in the position CYS-121, which is normally buried internally in the native molecule, is decisive for film formation [48,49,50,51]. Cohesion in films made out of native whey protein relies mainly on low energy bonding such as hydrogen bonding, electrostatic interactions and van der Waals forces [10].

The second most abundant whey protein α-lactalbumin is rich in lysine, leucine, threonine, tryptophan, and cysteine. The protein consists of 123 amino acids and has a molecular weight of about 14 kDA. The protein contains α-helix, β-sheet and random secondary structures (Figure 1). Each one of the eight cysteine residues is linked together whereby it contains four internal disulfide bridges. The protein α-lactalbumin is more heat stable than other whey proteins because of calcium bonds at the asparagine residues [45,49].

### 2.3. Soy Protein

Soy is a plant protein, which stores nitrogen and energy reserves for the germinating plant. It is a by-product from soybeans through an extraction process to obtain soy oil. Based on molecular weight and sedimentation coefficient, soy protein can be separated into 2S, 7S, 11S and 15S fractions [26,53,54].

Soy protein has high amounts of asparagine and glutamine residues. The major components of soy protein are the globular proteins β-conglycinin (7S fraction) and glycinin (11S fraction). Both fractions are tightly folded due to intramolecular cross-linking, though unstructured regions exist internally. The extent of intramolecular disulfide bridges is limited by only two to three cysteine per molecule β-conglycinin. Glycinin contains 20 disulfide bonds. The 7S protein, which makes up about 30% of the total protein, consists of three peptide subunits. The subunits are various combined and extensively glycosylated. The six acidic and basic subunits of glycinin (about 35% of the protein) are linked together via disulfide bonds [26,32,36,55,56,57,58,59].

### 2.4. Wheat Gluten

Wheat gluten (WG) is the byproduct, elastic mass after starch is washed from wheat flour dough. WG is an excellent film forming agent, but an addition of plasticizer to the film is necessary to form a homogenous film. Glutenins, gliadins, and the low molecular weight proteins albumins and globulins are the primary wheat protein fractions [27,60,61].

Gliadins can be classified into α-, β-, γ-, and ω-gliadins based on electrophoretic mobilities [62] at low pH and are characterized by their high content of glutamine residues. Hydrophobic interactions and intramolecular disulfide bonds cause the globular structure of gliadin. The terminal amino group of glutamine promotes hydrogen bonding among polypeptide chains. Glutenin is with a molecular weight over 1 × 10^7^, one of the largest natural polymeric molecules. Disulfide bonds are predominantly intermolecular in glutenin and contribute to viscoelastic properties of WG protein. The smaller globular gliadin polypeptides are packed into the network of the random-coiled glutenin polypeptide [18,27,31,60,62,63,64].

## 3. Cross-Linking in Protein Films

**Figure 2:**
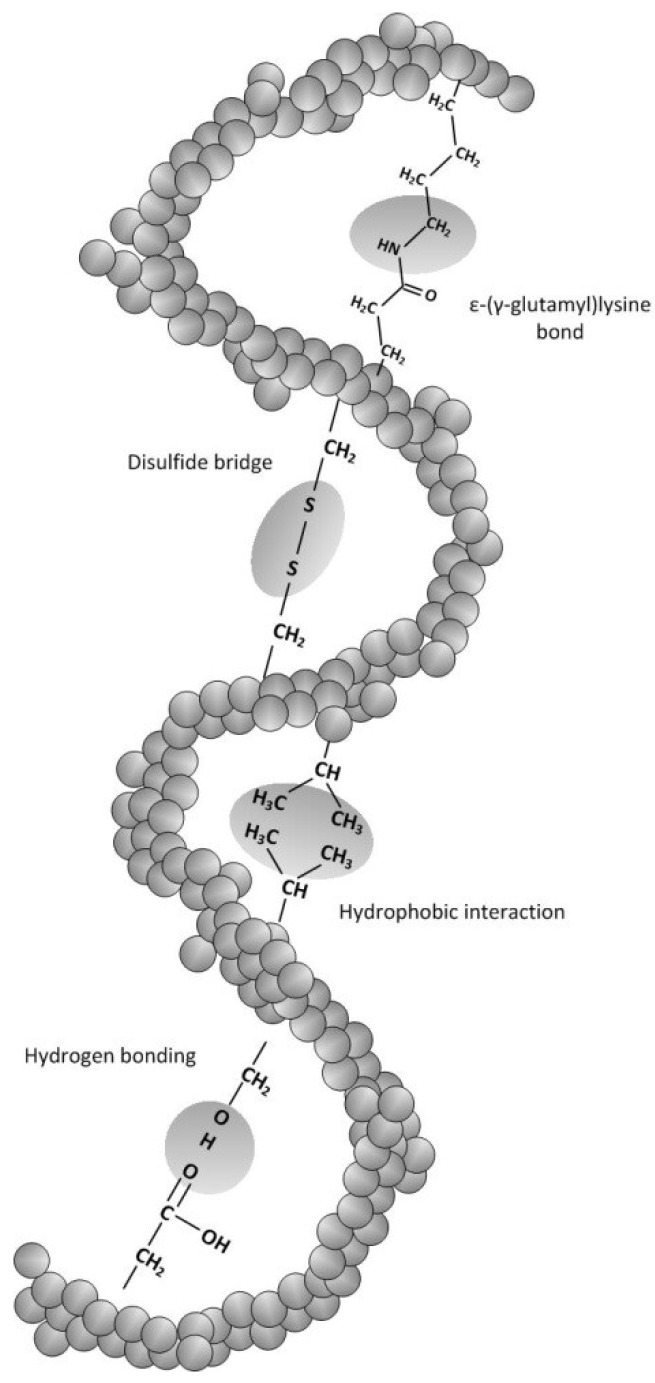
Schematic figure of possible inter-actions in cross-linked protein films. Adopted and extended from [67].

Cross-linking is a viable method to improve the mechanical strength and barrier properties against gas, vapors, and solutes by stronger intermolecular bonds, closer molecule packing and reduced polymer mobility. Figure 2 shows covalent and non-covalent interactions which can be formed after physical, chemical, and enzymatic treatments [65,66].

### 3.1. Thermal Cross-Linking

Exposing proteins to high temperatures leads in most cases to denaturation of the proteins. Heating changes the three-dimensional structure of proteins by disrupting hydrogen bonds and non-polar hydrophobic groups. Internal thiol and hydrophobic groups are exposed and can form intermolecular disulfide bond and hydrophobic interactions among the unfolded protein chains [37,68].

Pérez-Gago and Krochta [10] examined the impact of heating of the film forming properties of WPI solutions. The degree of denaturation of whey proteins was found to depend on the denaturation of β-lactoglobulin because it is the main component of whey proteins. According to de Wit [50], thermal denaturation of β-lactoglobulin occurs at 78 °C in a 0.7 M phosphate puffer (pH 6). Besides polymerization, newly-exposed groups can interact through covalent intermolecular disulfide bonds and non-covalent forces including hydrophobic, van der Waals and hydrogen interactions. The degree of protein unfolding and denaturation, caused by variation in heating time and temperature, influence the degree of protein cross-linking. Films made from heated whey protein solution revealed significantly lower oxygen permeability than that of films made from unheated solution [10,35,38,69,70].

The denaturation behavior of soy protein is affected by its major components. Glycinin has a denaturation temperature between 85 and 95 °C and due to the disulfide bonds in its structure is more heat stable than β-conglycinin (temperature of denaturation between 65 and 75 °C). Heating of soy protein forms soluble aggregates between the subunits of β-conglycinin and the polypeptide chain of glycinin [71,72,73,74]. Gennadios *et al*. [9] investigated the effect of pH of WG and soy protein film-forming solutions and hypothesized that covalent disulfide bonds are the main forces in film formation of dried film. Results of Stuchell and Krochta [75] showed that heat-treated soy protein films have lower water vapor permeability and higher tensile strength than the non-heat treated samples.

Heat treatment of film forming wheat gluten solutions leads to both formation of disulfide bonds from free thiol groups and disulfide-disulfide interchange reaction. Resulting wheat gluten aggregates are stabilized by irreversible cross-linking [31,76,77]. Besides solution-casting, processing techniques such as compression molding and extrusion gain in importance. Singh *et al*. [77] reported that gliadin polymerization occurs from above 90 °C and thus gluten materials are usually processed above 90 °C [13,61,77,78,79].

According to several authors, the higher the processing temperature the greater is the amount of protein cross-linking taking place. However, it must be considered that there is an upper limit of the processing window because too high a temperature can cause protein degradation and disruption of covalent bonds [32,36,78,80].

### 3.2. Enzymatic Cross-Linking

Enzymes can also be used to cross-link proteins. Transglutaminase is the most common enzyme to create protein cross-linkages. Transglutaminase equates to a protein-glutamine-γ-glutamyl transferase and catalyzes the acyl transfer reaction between the carboxyl amide group (acyl donor) of glutamine residues and the amino group (acyl acceptor) of lysine residues. The results are intra- and intermolecular covalent cross-linked proteins. The resulting ε-(γ-glutamyl)lysine bonds lead to high molecular weight polymers. WPI films with transglutaminase-crosslinked have improved properties. For example, both the oxygen permeability and water vapor transmission rate of whey protein layers were reduced by the use of transglutaminase [20,21,81,82,83,84,85,86,87].

Transglutaminase is now widely used. As an example, Motoki *et al*. [88,89] discovered the formation of heterologous biopolymers combining soy protein and casein through transglutaminase. HPLC and SDS-polyacrylamide gel electrophorese confirmed that the formation of ε-(γ-glutamyl)lysine bonds between the glutamyl residues of casein and the lysyl residues of the native soy protein is catalyzed by transglutaminase. A formation of cross-linked biopolymers from 11S soy protein fraction and whey protein has also been reported [86,90]. Advantages of ε-(γ-glutamyl)-lysyl cross-links are that the enzymatic treatment leads to greater tensile strength and lower solubility of the films [86].

The contrary was observed in cross-linking by horseradish peroxidase. Horseradish peroxidase is available in a few forms and acts on proteins in the presence of oxygen and in the absence of low molecular weight hydrogen donors. This enzyme catalyzes the oxidation of tyrosine amino acid residues and the formation of di-, tri-, and tetra-tyrosine. These products eventually promote protein cross-linking. Horseradish peroxidase increases the solubility in soy protein solutions and causes some degradation of the protein chains. The moisture barrier could not be improved and the final films were also extremely brittle [75].

Wheat gluten film-forming solutions are well suited for enzymatic cross-linking by transglutaminase because of the high content of glutamine residues. Transglutaminase catalyzes a protein cross-linking reaction through acyl transfer between glutamine residues and primary amines. The content of ε-(γ-glutamyl)lysine cross-links can be increased by heating, as heating increases the content of glutamine and lysine residues on the surface [91,92].

### 3.3. Irradiation

In general cross-linking by irradiation can be divided into two types, ionizing radiation (e.g., γ-irradiation) and non-ionizing irradiation (e.g., UltraViolet (UV) light). Ionizing irradiation can cause irreversible changes of protein conformation, oxidation of amino acids, breakage of covalent bonds, formation of protein free radicals, and recombination and polymerization reactions. The basic principle is that water forms hydroxyl radicals when exposed to γ-irradiation. Proteins with a high amount of aromatic amino acids, such as phenylalanine and tyrosine, react more with the hydroxyl radicals than with aliphatic amino acids [93]. The effect of γ-irradiation on the protein conformation depends on several factors, such as protein concentration, the presence of oxygen, and the quaternary structure of proteins [94]. Brault *et al*. [95] demonstrated that γ-irradiation forms bityrosine bridges between protein chains in milk protein films. Bityrosine bridges lead to an increase in molecular weight. Advantages of γ-irradiation in contrast to enzymatic cross-linking are that the process is less expensive and γ-irradiation allows the formation of insoluble and sterilizable films [95,96].

The ability to form bityrosine bridges in whey protein films is limited by the low content of tyrosine residues in β-lactoglobulin [40,97,98]. The γ-irradiated wheat gluten films have increased water vapor barrier properties, increased tensile strength, and decreased elongation at an irradiation dose of 10 kGy (kilogray is a well-known unit of irradiation dose), while higher doses lead to reduced effects [13,99,100]. UV radiation is the formation of covalent cross-links in proteins by recombination and UV absorption of the aromatic acid, such as tyrosine and phenylalanine [101,102]. Gennadios *et al*. [103] used UV radiation for cast SPI films. The UV irradiated films showed an increase in tensile strength and a reduction of elongation, which could be explained by a high amount of tyrosine and phenylalanine in the soy protein. UV-treatment had no effect on film water vapor permeability [13,103].

### 3.4. Chemical Cross-Linking

The functional properties can also be influenced by chemical cross-linking of protein molecules. Chemical treatment of soy protein and treatment of the film-forming whey protein solution were examined. Brandenburg *et al*. [14] applied alkali-treatment with 0.1 N NaOH on soy protein isolate powder. The treatment caused an increase in elongation of the resulting films. The solubilization and partially denaturation have no significant influence on tensile strength, water- and oxygen permeability but a higher e-modulus was reported. Chemical cross-linkers such as glutaraldehyde, glycal, formaldehyde, dialdehyde starch, and carbonyldiimidazole leads to increasing tensile strength, decreased elongation, reduced water solubility, decreased water vapor barrier, and increased oxygen barrier of the films [14,104,105].

## 4. Methods to Determine the Degree of Cross-Linking in Protein Films

In principle, the analytical methods rely on analysis of proteins in solutions and of solid protein films. The disadvantage of studying solutions is that interactions between proteins and solvents change the protein conformation and initial cross-linking.

### 4.1. Structural Analysis of Solutions

#### 4.1.1. Electrophoretic Analysis

Sodium dodecyl sulfate-polyacrylamide gel electrophorese (SDS-PAGE) is the most commonly used system to fractionate proteins according to their molecular weight. The separation medium is a discontinuous gel on the basis of polyacrylamide, and an electric field is applied across the gel, causing the negatively charged proteins to migrate across the gel towards the anode. Changes in the molecular masses of the proteins can be identified by SDS. SDS cleaves non-covalent linked aggregates into monomers, whereas covalent disulfide bridges remain intact. The longer the proteins are, the more they are retained in the gel. Following electrophoresis, the gel may be stained with coomassie brilliant blue to make the separated proteins visible. The evaluation can be done qualitatively with a plotted molecular weight size marker of known molecular weight [106].

Monahan *et al*. [107] defined polymerization as the appearance of high molecular weight protein bands in the stacking gel and at the top of the resolving gel with a concomitant decrease in the intensity of monomeric protein bands. Bands of aggregates in electrophoretic patterns were detected in both heated and irradiated whey proteins [84,107,108,109]. The immobile bands in the electrophoretic patterns are a sign of development of covalent intermolecular disulfide bonds induced by heating or by irradiation. Le Tien *et al*. [108] explained the accumulation caused by cross-linking of cysteine and the immobility of aromatic side chains. Heated β-lactoglobulin solutions were analyzed by SDS-PAGE to confirm that disulfide-linked aggregates are formed during heat treatment. Non-covalently linked protein aggregates are dispersed into monomers while aggregates linked through intermolecular disulfide-exchange reaction remain intact during electrophoretic analysis [110,111].

Stuchell *et al*. [75] used SDS-PAGE to identify changes in the conformation of soy protein in film-forming solution due to enzymatic treatment. The results show that distinct protein bands disappear from the gel during enzymatic treatment. Also smearing toward the top of the gel with increasing time of incubation is visible. Both indicate that enzymatic treatment causes cross-linking of protein molecules and partial degradation of protein chains [75,84,112].

Distinct bands at the molecular weights of gliadins and gluetnins are noticeable for native WG films. By heating at temperatures up to 75 °C these bands smear and most of the functionality of WG is destroyed [76,113].

Chawla *et al*. [114] and Le Tien *et al*. [108] used SDS-PAGE to verify formation of cross-linked proteins upon irradiation. SDS-PAGE pattern showed that the low molecular weight proteins (in the range of 14–20 kDa) were reduced upon irradiation and high molecular weight bands were observed. At irradiations doses of 60 kGy and above, high molecular mass aggregates fail to move into the stacking and separating gel. Therefore it can be concluded that the polymerization of whey protein upon irradiation is dose-dependent. The intensity of the aggregates bands increases with the UV dosage. UV radiation affects individual proteins in a different way due to varying amino acid compositions and molecular structure [103,108,114].

#### 4.1.2. Size Exclusion Chromatography

Size exclusion chromatography (SEC) is used to determine the molecular weight of control and cross-linked samples. The principle of SEC is that particles of different sizes will elute through a stationary phase at different rates. Small molecules will elute late, because they can enter every region of the stationary pore system. Large molecules will elute early.

Le Tien *et al*. [97] compared the size exclusion chromatography of control, heated and γ-irradiated whey protein isolate (WPI) and whey protein concentrate (WPC) solutions. The smallest molecular weight fraction can be attributed to native, intramolecular cross-linked proteins. As mentioned before, thermal treatment and irradiation lead to cross-linking via formation of new interactions and an increase of the molecular weight (see Table 2). Thus the molecular mass distribution increases for heated or irradiated WPI. However, the formation of high molecular mass aggregates by irradiation is limited by little content of tyrosine residues for cross-linking via bityrosine bridges [97,98,108].

**Table 2:** Molecular weight fractions of native, heated and γ-irradiated whey protein isolate (WPI) and WPC (whey protein concentrate) solutions [108].

Soluble fraction of film-forming solution	Molecular weight (kDa)	Responsible interactions
Native WPI/WPC	40	Native or intramolecular cross-linked
Heated WPI/WPC	600–3800	Disulfide bonds
γ-Irradiated WPI/WPC	1000–2000	Bityrosine bridges

Changes in the molecular weight distribution of cross-linked films based on soy protein [93] and wheat gluten [13,115] are also detected by SEC. The effect of cross-linking can also be derived from elution profile by enhancement of the molecular weight due to aggregation.

### 4.2. Structural Analysis of Solid Biofilms

#### 4.2.1. Spectroscopic Techniques

Circular dichroism and Fourier Transform Infrared analysis are used to determine changes at secondary structure level. Both can be applied to study protein conformational changes within films.

Circular dichroism (CD) spectroscopy. Circular dichroism is a specific characteristic of optically active molecules. Enantiomers of chiral substances absorb circularly polarized light in different ways. All amino acids (except glycine), α-helices and β-sheets are chiral and therefore optically active. Thus CD analysis is used to determine conformational changes in the secondary structure of proteins. Proteins do not unfold totally during gelation, a loss of α-helix structures and an increase in β-sheet can be observed. Changes in the CD spectrum indicate transconformation of α-helix and β-sheet structures to unordered structures after heating. The measured quantity is the ellipticity, which is caused by the asymmetry of the molecule [106,116,117,118].

A requirement to apply this analytical method for solid films is that the film samples must be transparent. This is problematic, because after processing treatment, protein films can darken in color, due to Maillard browning, or become hazy [119].

To our knowledge, CD is hardly ever used for structural analysis of soy protein or wheat gluten based films.

Fourier Transform Infrared (FTIR) spectroscopy. FTIR spectroscopy is used for characterization of protein secondary structure [120]. FTIR is highly versatile and can be used for samples of various states, including solutions, powders, and films [108,121]. Ambrose *et al*. [122] showed that the frequency of the so-called amide I and amide II absorptions of a protein is related to the secondary structure of the protein. While proteins contain different secondary structural elements (α-helix, β-sheet, β-turn and unordered structures) the amide I band is a composite band. The amide I absorptions relates to the C=O stretching mode of the protein backbone [32,108,120,123]. The molecular level of films made of whey protein [83,108,124], soy protein [32,73] and wheat gluten [125,126] have been investigated using FTIR.

Table 3 summarizes the spectral changes of exemplary whey protein based films. The studies confirm that thermal treatment leads to partial loss of the secondary structure and to protein aggregation by formation of intermolecular, β-sheet structures, and can be indicated by changes in the amide I region. If so, bands which indicate β-sheets and α-helix are minimized or eliminated and an additional band is formed as a result of the new arrangements [32,83,108,120,124,127].

**Table 3:** Structural changes in the amide I region of the Fourier Transform Infrared (FTIR) spectra of whey protein films in consequence of thermal denaturation [83,108].

Whey protein film	Wavenumber (cm^−1^)	Responsible interactions
Native	1621, 1634, 1692	Intramolecular β-sheets
	1649	α-helix
	1606, 1663, 1677	β-turns, side chain residues
Heated	Elimination and diminishment of the native bands	
	1612	Intermolecular β-sheets
	1682	Antiparallel β-sheets

Nuclear magnetic resonance spectroscopy (NMR). NMR is used to investigate the electronic environment of individual atoms and the interactions with their neighboring atoms. One- and two-dimensional NMR spectroscopy is commonly used to indicate changes in the conformation of proteins. The protein’s degree of unfolding can be determined by 1D-NMR, when resolved and exchangeable protons have exchanged with solvent deuterium. 2D-NMR gives information at still higher resolution. For example, ^1^H NMR is used to determine conformational changes occurring in β-LG during heating [128]. ^1^H NMR is very sensitive to conformational changes, reaches atomic resolution, and gives information on different parts of the protein. By heating, the secondary and tertiary structure will change due to breakage and reformation of intra- and intermolecular interactions. This should be shown up in changes in exchangeable amide proton resonance. The NMR spectrum of native β-LG shows that the peaks are broadened and poorly resolved. Whey protein gel formation, at temperatures above 70 °C, leads to peaks disappearance and decrease in peak intensity [106,128,129,130,131]. Literature research revealed that NMR has rarely been used for protein films until recently (particularly soy protein and wheat gluten films), but the method seems promising. In addition, NMR was quite recently used to evaluate the acylation of WPI via fatty acid vinyl esters [132].

#### 4.2.2. X-ray Scattering Methods

Native proteins exhibit crystallinity, which may be lost in the denaturation step of film formation because of unfolding of the amino acid chains. However, crystallinity can be rebuilt during drying because new interactions are formed. The presence and extent of crystallinity can be determined by X-ray scattering methods [133]. Le Tien *et al*. [108] indicated irreversible denaturation of whey proteins in the gelation process by X-ray diffraction. X-ray diffraction analysis showed that the formation of disulfide bonds between protein molecules by heating or γ-irradiation induces modification of the conformation of proteins. Proteins in whey protein films become more ordered and more stable [106,108,134].

Also small- and wide-angle X-ray scattering were used to gather information on the morphology and crystallinity of whey protein [127,135], soy protein [136], and wheat gluten [137,138] solutions. X-ray scattering results show to what extent the film forming process changes the internal structure and the film morphology.

## 5. Quantification of Degree of Cross-Linking in Protein Films

### 5.1. Swelling

Polymeric networks cannot be dissolved in fluid media, they swell instead. The degree of swelling depends on the interactions between the fluid and the polymer and on the structure and properties of both the solvent and the polymer. A higher degree of cross-linking decreases the swellability [139]. The fluid dissolves the network, while the elastic forces of the polymer counteract swelling. At a high degree of swelling, the entropy of the whole system decreases. The result of the swelling process is an equilibrium state. This state of swelling occurs when the chemical potential of the solvent in the polymer network equals the chemical potential of the free solvent.

The degree of cross-linking (ρ_c_) can be defined as the number of cross-linked monomers relative to the total number of monomers in a certain quantity of substance. This definition does not apply to practical systems well, because it does not involve free polymer chains or loops. Hence, instead of determining the number of linked monomers, swelling is an effective indirect method for determining the real degree of cross-linking. The degree of swelling (*Q*) can be calculated by comparing the weight of the swollen network (*a*) to the weight of the original network (*b*):

(1) $$Q=\;\frac{a-b}{b}$$

The reciprocal value (1/*Q*) is a relative value for the degree of cross-linking [70,140,141].

### 5.2. Protein Solubility Study

The functional groups determine the protein solubility and this suggest that cross-linking has a notable impact on the solubility of proteins. Therefore the protein solubility study is suited to determine the important bonds in cross-linking process. The procedure is based on protein solubilization by using different solvents. The selective reagents are capable of destroying hydrogen bonds and hydrophobic interactions and capable of splitting disulfide interactions [106,142].

Several studies reported that thermal and enzymatic treatment and UV-radiation lower the solubility of films made out of soy protein [32,112,142,143] and wheat gluten [125]. Liu *et al*. [144] improved the protein solubility study to investigate the importance of non-covalent and covalent bonds in the thermoplastic process of proteins. Different types of extractions reagents are used to determine the solubility of the extrudates.

Table 4 gives an overview of types of interactions and reagents able to break the interactions. The general salt buffer, such as phosphate buffer can extract protein in its native state. Urea or SDS break non-covalent interactions and the reducing agent, such as DTT, disrupts disulfide by thiol-disulfid-replacement. The procedure of the solubility study is as follows: (1) a buffered solution is made of all reagents to break all possible bonds; (2) one or more reagents are subtracted from the all containing buffered solution; and (3) the solubility values are compared with the all containing buffered solution. As a result, protein-protein interactions can be evaluated. In comparison with earlier literature, Liu *et al.* [142] used three reagents (thiourea, Triton X-100, CHAPS) to differentiate the relative importance among non-covalent interactions. Thiourea disrupts hydrophobic bonds more effectively, urea, however, is appropriate to hydrogen bonding [142,144,145].

The quantification can be carried out by Bradford-Assay. It is a rapid and sensitive photometric method for determination of protein concentrations which involves the dye Coomassie Brilliant Blue G-250. In presence of proteins, the red, cationic form of Coomassie Brilliant Blue G-250 changes to the blue, anionic form. Thus the absorption maximum of the protein increases from 465 to 595 nm by binding of the before mentioned dye and the protein concentration can be measured at 595 nm [146,147].

**Table 4:** Types of interactions, specific interactions and reagents able to break up the interactions [142].

Type of Interaction	Specific Interaction	Reagents Capable of Breaking up the Interactions
Covalent	Disulfide bonding	Oxidizing or reducing agents, e.g., performic acid, DTT
Non-Covalent	Hydrogen bonding	Strong H-bonding agents, e.g., urea, diemethyl formamide, thiourea, SDS
Non-Covalent	Hydrophobic interaction	Ionic and nonionic detergents, e.g., SDS, thiourea, Triton, CHAPS sodium salts of long-chain fatty acids
Non-Covalent electrostatic	Acid hydrophilic basic hydrophilic	Acids, alkali or salt solution

SDS, sodium dodecyl sulfate; DTT, dithiotreitol; CHAPS, (3-[(3-Cholamidopropyl)-dimethylammonio]-propan- sulfonat).

Table 5 gives an overview of the described methods and their use for solutions and films.

**Table 5:** Summary of qualification and quantification methods mentioned in this review of molecular interactions in protein based films and solutions.

State	Qualification	Quantification
Solution	SDS-Page Size exclusion chromatography X-ray scattering	–
Film	Spectroscopic techniques X-ray diffraction	Swelling protein solubility study

## 6. Conclusions

Protein based films have a promising potential to fulfill customer demands and expectations of new packaging systems that are biodegradable and made from renewable sources. However, mechanical and barrier properties still need to be improved, so that they are competitive with standard barrier polymers used today. The amino acid sequence of the protein is crucial for possible intermolecular cross-linking. Heat denaturation, enzymatic, chemical treatment, or irradiation can result in more stable films with improved barrier properties.

This work gives an overview of methods to determine and quantify intermolecular cross-links. The qualitative determination methods can be differentiated into structural analysis of solutions and of solid films. SDS-PAGE and size exclusion chromatography identify intermolecular cross-linking by increase of the molecular weight by aggregation. Spectroscopic techniques (CD, FTIR, NMR) characterize the secondary structure of proteins. It can be confirmed, that polymerization results in partial loss of the secondary structure and formation of new inter-molecular cross-links. Up to now CD and NMR have been rarely used for structural application of whey protein, soy protein and wheat gluten based film but they seem fairly promising. X-ray scattering methods such as WAXS (wide-angle X-ray scattering), are used to determine the crystallinity of proteins, while SAXS (small-angle X-ray Scattering) is common for studying the macromolecular structure of proteins, and thus the morphology of films. Qualitative determination methods have been more investigated than the quantitative determination of protein cross-linking. However, the degree of cross-linking can be calculated by the degree of swelling. The most promising quantitative method is the protein solubility study. Detailed information on specific interactions is given by studying the protein resolubilization by selective regents with several mechanisms of protein solubilization. An advantage of this method is that it can also be applied to the raw film material. Until now, the formation of new disulfide bonds as a result of cross-linking has only been detected in aqueous solutions and not in dry films [148]. Thus the protein solubility study would be suited to characterization of cross-linking in protein films and coatings. Hydrogen bonds, hydrophobic and covalent interactions could be quantified by this method.

## References

[1] Sängerlaub S., Gibis D., Kirchhoff E., Tittjung M., Schmid M., Müller K. Compensation of pinhole defects in food packages by application of iron-based oxygen scavenging multilayer films. Proceedings of the 5th international Symposium on Food Packaging.

[2] Buchner N. (1999). Verpackung von Lebensmitteln.

[3] Robertson G.L. (2006). Food Packaging: Principles and Practice.

[4] Cuq B., Gontard N., Guilbert S. (1998). Proteins as agricultural polymers for packaging production. Cereal Chem..

[5] Krochta J.M., Baldwin E.A., Nisperos-Carriedo M.O. (1994). Edible Coatings and Films to Improve Food Quality.

[6] Krochta J.M. (2002). Proteins as raw materials for films and coatings: definitions, current status, and opportunities. Protein-Based Films and Coatings.

[7] Gennadios A. (2002). Protein-Based Films and Coatings.

[8] Gennadios A., Weller C.L., Testin R.F. (1993). Modification of physical and barrier properties of edible wheat gluten-based films. Cereal Chem..

[9] Gennadios A., Brandenburg A.H., Weller C.L., Testin R.F. (1993). Effect of pH on properties of wheat gluten and soy protein isolate films. J. Agric. Food Chem..

[10] Perez-Gago M.B., Nadaud P., Krochta J.M. (1999). Water vapor permeability, solubility, and tensile properties of heat-denatured versus native whey protein films. J. Food Sci..

[11] Sothornvit R., Krochta J.M. (2000). Plasticizer effect on oxygen permeability of beta-lactoglobulin films. J. Agric. Food Chem..

[12] Sothornvit R., Krochta J.M. (2001). Plasticizer effect on mechanical properties of beta-lactoglobulin films. J. Food Eng..

[13] Micard V., Belamri R., Morel M.H., Guilbert S. (2000). Properties of chemically and physically treated wheat gluten films. J. Agric. Food Chem..

[14] Brandenburg A.H., Weller C.L., Testin R.F. (1993). Edible films and coatings from soy protein. J. Food Sci..

[15] Schmid M., Hinz L.-V., Wild F., Noller K. (2013). Effects of hydrolysed whey proteins on the techno-functional characteristics of whey protein-based films. Materials.

[16] Bugnicourt E., Schmid M., Nerney O.M., Wildner J., Smykala L., Lazzeri A., Cinelli P. (2013). Processing and validation of whey-protein-coated films and laminates at semi-industrial scale as novel recyclable food packaging materials with excellent barrier properties. Adv. Mater. Sci. Eng..

[17] Hernandez-Izquierdo V.M., Reid D.S., McHugh T.H., Berrios J.De.J., Krochta J.M. (2008). Thermal transitions and extrusion of glycerol-plasticized whey protein mixtures. J. Food Sci..

[18] Gennadios A., Weller C., Testin R. (1993). Temperature effect on oxygen permeability of edible protein‐based films. J. Food Sci..

[19] Schmid M., Hammann F., Winkler H. (2014). Technofunctional properties of films made from ethylene vinyl acetate/whey protein isolate compounds. Packag. Technol. Sci..

[20] Schmid M., Dallmann K., Bugnicourt E., Cordoni D., Wild F., Lazzeri A., Noller K. (2012). Properties of whey protein coated films and laminates as novel recyclable food packaging materials with excellent barrier properties. Int. J. Polym. Sci..

[21] Schmid M., Sängerlaub S., Wege L., Stäbler A. (2014). Properties of transglutaminase crosslinked whey protein isolate coatings and cast films. Packag. Technol. Sci..

[22] Schmid M., Müller K., Sängerlaub S., Stäbler A., Starck V., Ecker F., Noller K. (2014). Mechanical and barrier properties of thermoplastic whey protein isolate/ethylene vinyl acetate blends. J. Appl. Polym. Sci..

[23] Schmid M., Krimmel B., Grupa U., Noller K. (2014). Effects of thermally induced denaturation on technological-functional properties of whey protein isolate-based films. J. Dairy Sci..

[24] Cinelli P., Schmid M., Bugnicourt E., Wildner J., Bazzichi A., Anguillesi I., Lazzeri A. (2014). Whey protein layer applied on biodegradable packaging film to improve barrier properties while maintaining biodegradability. Polym. Degrad. Stab..

[25] Kinsella J., Whitehead D. (1989). Proteins in whey: Chemical, physical, and functional properties. Adv. Food Nutr. Res..

[26] Kinsella J.E. (1979). Functional properties of soy proteins. J. Am. Oil Chem. Soc..

[27] Kinsella J. (1982). Relationships between structure and functional properties of food proteins. Food Proteins.

[28] Belitz H.-D.G.W., Schieberle P. (2008). Lehrbuch der Lebensmittelchemie: Mit 634 Tabellen.

[29] Cheftel J., Cuq J., Lorient D. (1985). Amino acids, peptides, and proteins. Food Chem..

[30] Panyam D., Kilara A. (1996). Enhancing the functionality of food proteins by enzymatic modification. Trends Food Sci. Technol..

[31] Hernández-Muñoz P., Villalobos R., Chiralt A. (2004). Effect of thermal treatments on functional properties of edible films made from wheat gluten fractions. Food Hydrocoll..

[32] Ciannamea E.M., Stefani P.M., Ruseckaite R.A. (2014). Physical and mechanical properties of compression molded and solution casting soybean protein concentrate based films. Food Hydrocoll..

[33] Barone J.R., Dangaran K., Schmidt W.F. (2006). Blends of cysteine-containing proteins. J. Agric. Food Chem..

[34] Were L., Hettiarachchy N., Coleman M. (1999). Properties of cysteine‐added soy protein‐wheat gluten films. J. Food Sci..

[35] Pérez‐Gago M., Nadaud P., Krochta J. (1999). Water vapor permeability, solubility, and tensile properties of heat‐denatured versus native whey protein films. J. Food Sci..

[36] Hernandez-Izquierdo V.M., Krochta J.M. (2008). Thermoplastic processing of proteins for film formation—A review. J. Food Sci..

[37] Damodaran S. (1996). Amino acids, peptides, and proteins. Food Sci. Technol..

[38] Kinsella J., Whitehead D., Brady J., Bringe N., Fox P. (1989). Milk proteins: Possible relationships of structure and function. Developments in Dairy Chemistry. 4. Functional Milk Proteins.

[39] Zheng H.-G., Yang X.-Q., Ahmad I., Min W., Zhu J.-H., Yuan D.-B. (2009). Soybean β-conglycinin constituent subunits: Isolation, solubility and amino acid composition. Food Res. Int..

[40] Etzel M.R. (2004). Manufacture and use of dairy protein fractions. J. Nutr..

[41] Lasztity R. (1995). The Chemistry of Cereal Proteins.

[42] Foegeding E.A., Mleko S.W. (2002). Whey protein products. Encyclopedia of Dairy Sciences.

[43] Jovanovic S., Barac M., Macej O. (2005). Whey proteins-properties and possibility of application. Mljekarstvo.

[44] Yada R.Y. (2004). Proteins in Food Processing.

[45] De Wit J. (1990). Thermal stability and functionality of whey proteins. J. Dairy Sci..

[46] Dybing S., Smith D. (1991). Relation of chemistry and processing precedures to whey protein functionality: A review. Cult. Dairy Prod. J..

[47] Belitz H.-D., Grosch W., Schieberle P. (2009). Food Chemistry: With 634 Tables.

[48] Sawyer W.H., Norton R.S., Nichol L.W., McKenzie G.H. (1971). Thermodenaturation of bovine beta-leactoglobulin kinetics and introduction of beta-structure. Biochim. Biophys. Acta.

[49] Töpel A. (2004). Chemie und Physik der Milch: Naturstoff-Rohstoff-Lebensmittel.

[50] De Wit J.N. (2001). Lecturer’s Handbook on Whey and Whey Products.

[51] Morr C., Ha E. (1993). Whey protein concentrates and isolates: Processing and functional properties. Crit. Rev. Food Sci. Nutr..

[52] Laboratory E.M.B. Protein Data Bank in Europe 2014. http://www.ebi.ac.uk/pdbe.

[53] Cho S.Y., Rhee C. (2004). Mechanical properties and water vapor permeability of edible films made from fractionated soy proteins with ultrafiltration. LWT Food Sci. Technol..

[54] Kumar R., Liu D., Zhang L. (2008). Advances in proteinous biomaterials. J. Biobased Mater. Bioenergy.

[55] Kunte L., Gennadios A., Cuppett S., Hanna M., Weller C.L. (1997). Cast films from soy protein isolates and fractions 1. Cereal Chem..

[56] Iwabuchi S., Yamauchi F. (1987). Electrophoretic analysis of whey proteins present in soybean globulin fractions. J. Agric. Food Chem..

[57] Hermansson A. (1978). Physico-chemical aspects of soy proteins structure formation. J. Texture Stud..

[58] Mori T., Utsumi S., Inaba H., Kitamura K., Harada K. (1981). Differences in subunit composition of glycinin among soybean cultivars. J. Agric. Food Chem..

[59] Utsumi S., Kinsella J.E. (1985). Forces involved in soy protein gelation: effects of various reagents on the formation, hardness and solubility of heat‐induced gels made from 7S, 11S, and soy isolate. J. Food Sci..

[60] Lasztity R. (1986). Recent results in the investigation of the structure of the gluten complex. Food Nahrung.

[61] Lagrain B., Goderis B., Brijs K., Delcour J.A. (2010). Molecular basis of processing wheat gluten toward biobased materials. Biomacromolecules.

[62] Shewry P., Tatham A. (1997). Disulphide bonds in wheat gluten proteins. J. Cereal Sci..

[63] Wrigley C., Bietz J., Pomeranz Y. (1988). Proteins and amino acids. Wheat: Chemistry and Technology.

[64] Wieser H. (2007). Chemistry of gluten proteins. Food Microbiol..

[65] Avena-Bustillos R.J., Krochta J.M. (1993). Water vapor permeability of caseinate-based edible films as affected by pH, calcium crosslinking and lipid content. J. Food Sci..

[66] Kester J.J., Richardson T. (1984). Modification of whey proteins to improve functionality. J. Dairy Sci..

[67] Verbeek C.J.R., van den Berg L.E. (2010). Extrusion processing and properties of protein-based thermoplastics. Macromol. Mater. Eng..

[68] Shimada K., Cheftel J.C. (1989). Sulfhydryl group/disulfide bond interchange reactions during heat-induced gelation of whey protein isolate. J. Agric. Food Chem..

[69] Kinsella J.E., Morr C.V. (1984). Milk proteins: Physicochemical and functional properties. Crit. Rev. Food Sci. Nutr..

[70] Perez-Gago M.B., Krochta J.M. (2001). Denaturation time and temperature effects on solubility, tensile properties, and oxygen permeability of whey protein edible films. J. Food Sci..

[71] Wu S., Murphy P.A., Johnson L.A., Fratzke A.R., Reuber M.A. (1999). Pilot-plant fractionation of soybean glycinin and β-conglycinin. J. Am. Oil Chem. Soc..

[72] German B., Damodaran S., Kinsella J.E. (1982). Thermal dissociation and association behavior of soy proteins. J. Agric. Food Chem..

[73] Subirade M., Kelly I., Guéguen J., Pézolet M. (1998). Molecular basis of film formation from a soybean protein: Comparison between the conformation of glycinin in aqueous solution and in films. Int. J. Biol. Macromol..

[74] Renkema J.M., van Vliet T. (2002). Heat-induced gel formation by soy proteins at neutral pH. J. Agric. Food Chem..

[75] Stuchell Y.M., Krochta J.M. (1994). Enzymatic treatments and thermal effects on edible soy protein films. J. Food Sci..

[76] Schofield J., Bottomley R., Timms M., Booth M. (1983). The effect of heat on wheat gluten and the involvement of sulphydryl-disulphide interchange reactions. J. Cereal Sci..

[77] Singh H., MacRitchie F. (2004). Changes in proteins induced by heating gluten dispersions at high temperature. J. Cereal Sci..

[78] Gällstedt M., Mattozzi A., Johansson E., Hedenqvist M.S. (2004). Transport and tensile properties of compression-molded wheat gluten films. Biomacromolecules.

[79] Pommet M., Redl A., Morel M.H., Domenek S., Guilbert S. (2003). Thermoplastic processing of protein-based bioplastics: Chemical engineering aspects of mixing, extrusion and hot molding. Macromol. Symp..

[80] Sothornvit R., Olsen C.W., McHugh T.H., Krochta J.M. (2003). Formation conditions, water-vapor permeability, and solubility of compression-molded whey protein films. J. Food Sci..

[81] De Jong G.A.H., Koppelman S.J. (2002). Transglutaminase catalyzed reactions: Impact on food applications. J. Food Sci..

[82] Zhu Y., Rinzema A., Tramper J., Bol J. (1995). Microbial transglutaminase—A review of its production and application in food processing. Appl. Microbiol. Biotechnol..

[83] Eissa A.S., Puhl C., Kadla J.F., Khan S.A. (2006). Enzymatic cross-linking of β-lactoglobulin: Conformational properties using FTIR spectroscopy. Biomacromolecules.

[84] Truong V.D., Clare D.A., Catignani G.L., Swaisgood H.E. (2004). Cross-linking and rheological changes of whey proteins treated with microbial transglutaminase. J. Agric. Food Chem..

[85] Schmid M. (2013). Properties of cast films made from different ratios of whey protein isolate, hydrolysed whey protein isolate and glycerol. Materials.

[86] Yildirim M., Hettiarachchy N.S. (1998). Properties of films produced by cross-linking whey proteins and 11S globulin using transglutaminase. J. Food Sci..

[87] Oh J.-H., Wang B., Field P.D., Aglan H.A. (2004). Characteristics of edible films made from dairy proteins and zein hydrolysate cross-linked with transglutaminase. Int. J. Food Sci. Technol..

[88] Motoki M., Nio N., Takinami K. (1987). Functional properties of heterologous polymer prepared by transglutaminase between milk casein and soybean globulin. Agric. Biol. Chem..

[89] Motoki M., Seguro K. (1998). Transglutaminase and its use for food processing. Trends Food Sci. Technol..

[90] Yildirim M., Hettiarachchy N.S. (1997). Biopolymers produced by cross-linking soybean 11S globulin with whey proteins using transglutaminase. J. Food Sci..

[91] Wang J.-S., Zhao M.-M., Yang X.-Q., Jiang Y.-M., Chun C. (2007). Gelation behavior of wheat gluten by heat treatment followed by transglutaminase cross-linking reaction. Food Hydrocoll..

[92] Tseng C.S., Lai H.M. (2002). Physicochemical properties of wheat flour dough modified by microbial transglutaminase. J. Food Sci..

[93] Sabato S., Ouattara B., Yu H., D’aprano G., Le Tien C., Mateescu M., Lacroix M. (2001). Mechanical and barrier properties of cross-linked soy and whey protein based films. J. Agric. Food Chem..

[94] Yang Y.C., Song K.B. (1999). Effect of ascorbic acid and protein concentration on the molecular weight profile of bovine serum albumin and β-lactoglobulin by γ-irradiation. Food Res. Int..

[95] Brault D., D’Aprano G., Lacroix M. (1997). Formation of free-standing sterilized edible films from irradiated caseinates. J. Agric. Food Chem..

[96] Davies K., Delsignore M., Lin S. (1987). Protein damage and degradation by oxygen radicals. II. Modification of amino acids. J. Biol. Chem..

[97] Hoffmann M.A., Sala G., Olieman C., de Kruif K.G. (1997). Molecular mass distributions of heat-induced β-lactoglobulin aggregates. J. Agric. Food Chem..

[98] Wong D.W.S., Camirand W.M., Pavlath A.E. (1996). Structures and functionalities of milk proteins. Crit. Rev. Food Sci. Nutr..

[99] Köksel H., Sapirstein H., Celik S., Bushuk W. (1998). Effects of gamma-irradiation of wheat on gluten proteins. J. Cereal Sci..

[100] Lee S., Lee M., Song K. (2005). Effect of gamma-irradiation on the physicochemical properties of gluten films. Food Chem..

[101] Forbes W.F., Sullivan P.D. (1966). The effect of radiation on collagen I. Electron-spin resonance spectra of 2537-Å-irradiated collagen. Biochim. Biophys. Acta BBA Biophys. Incl. Photosynth..

[102] Fujimori E. (1965). Ultraviolet light‐induced change in collagen macromolecules. Biopolymers.

[103] Gennadios A., Rhim J., Handa A., Weller C., Hanna M. (1998). Ultraviolet radiation affects physical and molecular properties of soy protein films. J. Food Sci..

[104] Wihodo M., Moraru C.I. (2012). Physical and chemical methods used to enhance the structure and mechanical properties of protein films. A review. J. Food Eng..

[105] Ustunol Z., Mert B. (2004). Water solubility, mechanical, barrier, and thermal properties of cross-linked whey protein isolate-based films. J. Food Sci..

[106] Whitford D. (2005). Proteins: Structure and Function.

[107] Monahan F.J., German J.B., Kinsella J.E. (1995). Effect of pH and temperature on protein unfolding and thiol/disulfide interchange reactions during heat-induced gelation of whey proteins. J. Agric. Food Chem..

[108] Le Tien C., Letendre M., Ispas-Szabo P., Mateescu M.A., Delmas-Patterson G., Yu H.L., Lacroix M. (2000). Development of biodegradable films from whey proteins by cross-linking and entrapment in cellulose. J. Agric. Food Chem..

[109] Yildirim M., Hettiarachchy N.S., Kalapathy U. (1996). Properties of biopolymers from cross-linking whey protein isolate and soybean 11S globulin. J. Food Sci..

[110] Roefs S., Dekruif K.G. (1994). A model for the denautartion and aggregation of beta-lactoglobulin. Eur. J. Biochem..

[111] Hoffmann M.A.M., van Mil P. (1997). Heat-induced aggregation of beta-lactoglobulin: Role of the free thiol group and disulfide bonds. J. Agric. Food Chem..

[112] Rangavajhyala N., Ghorpade V., Hanna M. (1997). Solubility and molecular properties of heat-cured soy protein films. J. Agric. Food Chem..

[113] Roy S., Weller C., Gennadios A., Zeece M., Testin R. (1999). Physical and molecular properties of wheat gluten films cast from heated film‐forming solutions. J. Food Sci..

[114] Chawla S., Chander R., Sharma A. (2009). Antioxidant properties of Maillard reaction products obtained by gamma-irradiation of whey proteins. Food Chem..

[115] Redl A., Morel M.H., Bonicel J., Vergnes B., Guilbert S. (1999). Extrusion of wheat gluten plasticized with glycerol: Influence of process conditions on flow behavior, rheological properties, and molecular size distribution. Cereal Chem..

[116] Zhu H., Damodaran S. (1994). Heat-induced conformational changes in whey protein isolate and its relation to foaming properties. J. Agric. Food Chem..

[117] Wada R., Fujita Y., Kitabatake N. (2006). Effects of heating at neutral and acid pH on the structure of β-lactoglobulin A revealed by differential scanning calorimetry and circular dichroism spectroscopy. Biochim. Biophys. Acta BBA Gen. Subj..

[118] Qi X.L., Holt C., McNulty D., Clarke D.T., Brownlow S., Jones G.R. (1997). Effect of temperature on the secondary structure of beta-lactoglobulin at pH 6.7, as determined by CD and IR spectroscopy: A test of the molten globule hypothesis. Biochem. J..

[119] Kelly S.M., Jess T.J., Price N.C. (2005). How to study proteins by circular dichroism. Biochim. Biophys. Acta BBA Proteins Proteomics.

[120] Jackson M., Mantsch H.H. (1995). The use and misuse of FTIR spectroscopy in the determination of protein structure. Crit. Rev. Biochem. Mol. Biol..

[121] González A., Strumia M.C., Alvarez Igarzabal C.I. (2011). Cross-linked soy protein as material for biodegradable films: Synthesis, characterization and biodegradation. J. Food Eng..

[122] Ambrose E., Elliott A. (1951). The structure of synthetic polypeptides. II. Investigation with polarized infra-red spectroscopy. Proc. R. Soc. Lon. Ser. A Math. Phys. Sci..

[123] Zhao X., Chen F., Xue W., Lee L. (2008). FTIR spectra studies on the secondary structures of 7S and 11S globulins from soybean proteins using AOT reverse micellar extraction. Food Hydrocoll..

[124] Lefèvre T., Subirade M., Pézolet M. (2005). Molecular description of the formation and structure of plasticized globular protein films. Biomacromolecules.

[125] Mangavel C., Barbot J., Popineau Y., Guéguen J. (2001). Evolution of wheat gliadins conformation during film formation: A Fourier transform infrared study. J. Agric. Food Chem..

[126] Georget D.M., Belton P.S. (2006). Effects of temperature and water content on the secondary structure of wheat gluten studied by FTIR spectroscopy. Biomacromolecules.

[127] Panick G., Malessa R., Winter R. (1999). Differences between the pressure-and temperature-induced denaturation and aggregation of β-lactoglobulin A, B, and AB monitored by FT-IR spectroscopy and small-angle X-ray scattering. Biochemistry.

[128] Belloque J., Smith G.M. (1998). Thermal denaturation of β-lactoglobulin. A 1H NMR study. J. Agric. Food Chem..

[129] Li H., Hardin C.C., Foegeding E.A. (1994). NMR studies of thermal denaturation and cation-mediated aggregation of β-lactoglobulin. J. Agric. Food Chem..

[130] Molinari H., Ragona L., Varani L., Musco G., Consonni R., Zetta L., Monaco H.L. (1996). Partially folded structure of monomeric bovine β-lactoglobulin. FEBS Lett..

[131] Kavanagh G.M., Clark A.H., Ross-Murphy S.B. (2000). Heat-induced gelation of globular proteins: Part 3. Molecular studies on low pH β-lactoglobulin gels. Int. J. Biol. Macromol..

[132] Winkler H., Vorwerg W., Schmid M. (2015). Synthesis of hydrophobic whey protein isolate by acylation with fatty acids. Eur. Polym. J..

[133] Lent L., Vanasupa L., Tong P. (1998). Whey protein edible film structures determined by atomic force microscope. J. Food Sci..

[134] Lacroix M., Le T., Ouattara B., Yu H., Letendre M., Sabato S., Mateescu M., Patterson G. (2002). Use of γ-irradiation to produce films from whey, casein and soya proteins: Structure and functionals characteristics. Radiat. Phys. Chem..

[135] Arai M., Ikura T., Semisotnov G.V., Kihara H., Amemiya Y., Kuwajima K. (1998). Kinetic refolding of β-lactoglobulin. Studies by synchrotron X-ray scattering, and circular dichroism, absorption and fluorescence spectroscopy. J. Mol. Biol..

[136] Chen P., Zhang L. (2005). New evidences of glass transitions and microstructures of soy protein plasticized with glycerol. Macromol. Biosci..

[137] Thomson N.H., Miles M.J., Popineau Y., Harries J., Shewry P., Tatham A.S. (1999). Small angle X-ray scattering of wheat seed-storage proteins: α-, γ- and ω-gliadins and the high molecular weight (HMW) subunits of glutenin. Biochim. Biophys. Acta BBA Protein Struct. Mol. Enzymol..

[138] Kuktaite R., Plivelic T.S., Cerenius Y., Hedenqvist M.S., Gällstedt M., Marttila S., Ignell R., Popineau Y., Tranquet O., Shewry P.R. (2011). Structure and morphology of wheat gluten films: From polymeric protein aggregates toward superstructure arrangements. Biomacromolecules.

[139] Schmidt M., Rodler N., Miesbauer O., Rojahn M., Vogel T., Dörfler R., Kucukpinar E., Langowski H.-C. (2012). Adhesion and barrier performance of novel barrier adhesives used in multilayered high-barrier laminates. J. Adhes. Sci. Technol..

[140] Cluff E., Gladding E., Pariser R. (1960). A new method for measuring the degree of crosslinking in elastomers. J. Polym. Sci..

[141] Bigi A., Cojazzi G., Panzavolta S., Roveri N., Rubini K. (2002). Stabilization of gelatin films by crosslinking with genipin. Biomaterials.

[142] Liu K., Hsieh F.-H. (2008). Protein–protein interactions during high-moisture extrusion for fibrous meat analogues and comparison of protein solubility methods using different solvent systems. J. Agric. Food Chem..

[143] Rhim J.W., Gennadios A., Handa A., Weller C.L., Hanna M.A. (2000). Solubility, tensile, and color properties of modified soy protein isolate films. J. Agric. Food Chem..

[144] Liu K.S., Hsieh F.-H. (2007). Protein-protein interactions in high moisture-extruded meat analogs and heat-induced soy protein gels. J. Am. Oil Chem. Soc..

[145] Li M., Lee T.-C. (1996). Effect of extrusion temperature on solubility and molecular weight distribution of wheat flour proteins. J. Agric. Food Chem..

[146] Bradford M.M. (1976). A rapid and sensitive method for the quantitation of microgram quantities of protein utilizing the principle of protein-dye binding. Anal. Biochem..

[147] Compton S.J., Jones C.G. (1985). Mechanism of dye response and interference in the Bradford protein assay. Anal. Biochem..

[148] Floris R., Bodnar I., Weinbreck F., Alting A.C. (2008). Dynamic rearrangement of disulfide bridges influences solubility of whey protein coatings. Int. Dairy J..

